# The hot-spot p53R172H mutant promotes formation of giant spermatogonia triggered by DNA damage

**DOI:** 10.1038/onc.2016.374

**Published:** 2016-11-21

**Authors:** Y Xue, A Raharja, W Sim, E S M Wong, S A B Rahmat, D P Lane

**Affiliations:** 1 p53 Laboratory, A*STAR, Singapore; 2 Barts and the London School of Medicine and Dentistry, London, UK; 3 Institute of Medical Biology, A*STAR, Singapore

## Abstract

Overexpression of mutant p53 is a common finding in most cancers but testicular tumours accumulate wild-type p53 (wtp53). In contrast to the accepted concept that p53 homozygous mutant mice do not accumulate mutant p53 in normal cells, our study on a mutant p53 mouse model of Li-Fraumeni syndrome harbouring the hot-spot p53R172H mutation described an elevated level of mutant p53 in non-cancerous mouse tissues. Here we use detailed immunohistochemical analysis to document the expression of p53R172H in mouse testis. In developing and adult testes, p53R172H was expressed in gonocytes, type A, Int, B spermatogonia as well as in pre-Sertoli cells and Leydig cells but was undetectable in spermatocytes and spermatids. A similar staining pattern was demonstrated for wtp53. However, the intensity of wtp53 staining was generally weaker than that of p53R172H, which indicates that the expression of p53R172H can be a surrogate marker of p53 gene transcription. Comparing the responses of wtp53 and p53R172H to irradiation, we found persistent DNA double-strand breaks in p53R172H testes and the formation of giant spermatogonia (GSG) following persistent DNA damage in p53R172H and p53-null mice. Strikingly, we found that p53R172H promotes spontaneous formation of GSG in non-stressed p53R172H ageing mice. Two types of GSG: Viable and Degenerative GSG were defined. We elucidate the factors involved in the formation of GSG: the loss of p53 function is a requirement for the formation of GSG whereas DNA damage acts as a promoting trigger. The formation of GSG does not translate to higher efficacy of testicular tumorigenesis arising from mutant p53 cells, which might be due to the presence of delayed-onset of p53-independent apoptosis.

## Introduction

Testicular malignancy is the most common cancer type in males aged 15–34 years and is still on the rise.^1^ Despite p53 being mutated in 50% of cancer types, it is not necessary for testicular tumorigenesis.^2^ In fact, testicular cancer typically overexpresses wild-type p53 (wtp53) and perhaps as a result is very responsive to chemotherapy.^3, 4^ While there has been effort to explain how testicular cancer tolerates wtp53,^5^ little is known why testicular cancers do not harbour mutated p53. It is not clear why tumour cells with p53 mutations are not selected for in the development of this cancer.

The level of p53 protein is tightly regulated by Mdm2, which promotes proteasomal degradation of p53 to maintain its low basal level in the absence of cellular stressors. Our previous study on a mouse model of Li-Fraumeni syndrome with a missense mutation at codon 172 of the p53 gene (producing a structural mutant p53R172H) described an elevated level of p53R172H in cycling/proliferating but non-cancerous morphologically normal mouse tissues. Similar to wtp53, p53R172H is also degraded by Mdm2 and responds to irradiation.^6^ The ability to detect p53R172H protein in morphologically normal tissues provides an avenue to study the regulation of p53R172H expression throughout the different stages of mouse development under non-stressed conditions and in the presence of cellular stressors (for example irradiation), which further helps us to understand the function and distribution of wtp53 in normal mouse tissues.

Spermatogenesis progresses through a series of cycling cell lineage development relying on the activities of spermatogonial stem cells and transient amplifying spermatogonia.^7, 8, 9^ Testicular cells are sensitive to DNA damage; thus, the testis is a suitable organ to study expression patterns of p53R172H and its response to DNA damage. The progression of spermatogonial stem cells passes through 12 stages of seminiferous epithelial cycles,^10^ with each stage being characterized by a combination of testicular cell types. Residing on the basement membrane of seminiferous tubules, undifferentiated spermatogonia (A_undiff_ or Asg) proliferate to form differentiating spermatogonia (A_diff_) type A1 at stage VIII, and type A2, A3, A4, Int, B spermatogonia during stage IX–VI. A_undiff_ become quiescent at stages III–VII. Inactivated preleptotene spermatocytes can be found in a more adluminal position at stage VII, and further differentiate to active preleptotene spermatocytes at stage VIII, which enter the prophase of meiosis (leptotene and zygotene) at stages IX–XII.^11, 12^ In addition, there are supportive cells, for example Leydig cells that reside at highly vascularized interstitium, and Sertoli cells, which are in close proximity to spermatogonia and basal lamina.

In this study, we investigated the expression of p53 proteins – wtp53 and p53R172H mutant (mutp53) through a detailed immunohistochemistry (IHC) analysis of morphologically normal embryonic, postnatal and adult mouse testis. We looked for any difference in the way mutant testicular cells respond to DNA damage induced by γ-irradiation. We also studied the effect of loss of p53 function using *p53*^*−/−*^ mice and compared this to *p53*^*R172H/R172H*^ mice to check for any gain-of-function of p53R172H. We are particularly interested in the formation and clearance of giant spermatogonia (GSG), which might be associated with subsequent development^13^ or elimination of testicular malignancy after p53 mutation.

## Results

### Expression of mutant p53 in morphologically normal *p53*^*R172H/R172H*^ mouse testis

**Figure 1:**
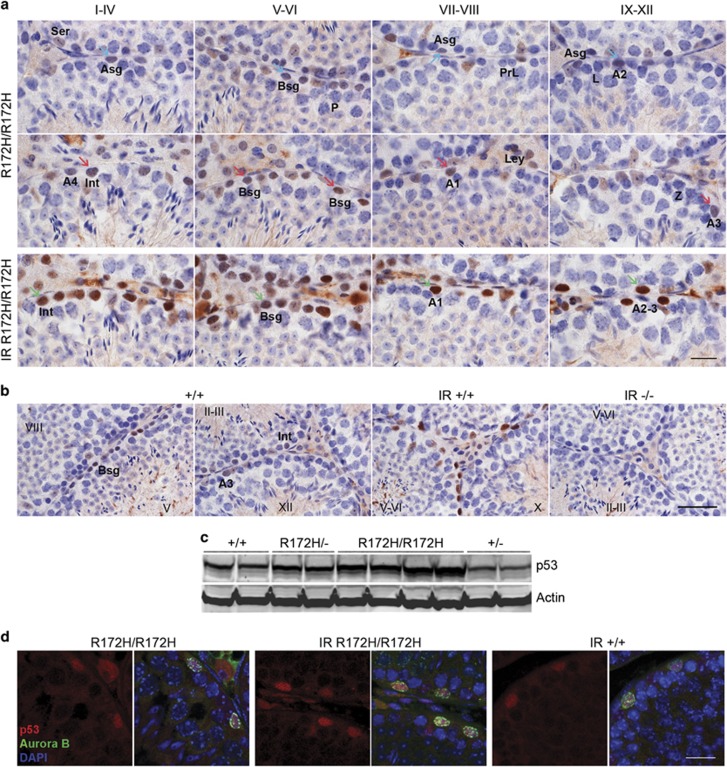
Mutant p53 expression in various spermatogonia and subset of Leydig cells. (**a**) p53 IHC of unirradiated and irradiated (8 Gy) testes from adult *p53*^*R172H/R172H*^ mice. The stages of the seminiferous epithelium cycles are indicated with Roman numerals. Brown nuclear staining indicates positive for mutp53. Examples marked with cyan arrows (low mutp53), red arrows (high mutp53) and green arrows (irradiation induced mutp53 expression). Scale bar=20 μm. (**b**) p53 IHC of testes from adult *p53*^*+/+*^ and *p53*^*−/−*^ mice. p53 stained in *p53*^*+/+*^ irradiated mouse testis as a positive control. p53 stained in *p53*^*−/−*^ irradiated mouse testis as a negative control. Scale bar=50 μm. In both (**a** and **b**): IR: 24 h post 8 Gy irradiation. Ser: Sertoli cell, A1-4: type A1-4 spermatogonia, Asg: type A_undiff_ spermatogonia, Bsg: type B spermatogonia, Int: intermediate spermatogonia, P: pachytene, PrL: preleptotene, L: leptotene, Z: zygotene, Ley: Leydig cell. (**c**) Western blot analysis with p53 antibody to detect endogenous wild-type and mutant p53 in testes of various p53 genotypes. Actin was detected as a loading control. (**d**) p53/Aurora B co-labelling of testes from *p53*^*R172H/R172H*^ mice without irradiation (R172H/R172H), with 8 Gy irradiation (IR R172H/R172H) and from *p53*^*+/+*^ (IR +/+) mice with 8 Gy irradiation. Scale bar=20 μm.

*p53*^*R172H/R172H*^ mice exhibit morphologically normal testes with well-developed seminiferous tubules and interstitium. There was detectable and heterogeneous mutp53 expression in spermatogonia and Leydig cells in *p53*^*R172H/R172H*^ mice (Figure 1a). We analysed the stages of seminiferous tubules to identify the specific types of p53-positive spermatogonia. A_undiff_ and A1-A4 spermatogonia were identified by their location at basal lamina and their ovoid nucleus, showing little or no heterochromatin.^14, 15^ Expression of mutp53 in quiescent A_undiff_ was low or undetectable and could be found in various stages of seminiferous tubules (Figure 1a, Asg). Proliferating A1-4 (in stage VIII–I) and Int spermatogonia (in stages I–IV) showed obvious mutp53 staining. Highest mutp53 expression was observed in a subset of type B spermatogonia (Bsg) (in stages V–VI). Sertoli cells show weak (almost negligible) mutp53 expression. Mutp53 was undetectable in primary spermatocytes, spermatids and myoid cells.

It was noted that *p53*^*+/+*^ testes showed detectable wtp53 levels similarly in Bsg, Int and A_diff_ spermatogonia (Figure 1b). The intensity of wtp53 staining was generally weaker than that of mutp53. However, since staining patterns of wtp53 and mutp53 are similar, mutp53 expression can be a surrogate marker of p53 gene transcription. The levels of p53 protein in distinct genotypes were further validated by western blot analysis. The amount of p53 protein was in the order of *p53*^*R172H/R172H*^ *> p53*^*R172H/−*^*>p53*^*+/+*^ *> p53*^*+/−*^ in a gene-dosage dependent manner (Figure 1c, Supplementary Figure S1).

Similar to wtp53 responses to irradiation, the levels of mutp53 were also increased. The mutp53 expression patterns remained cell specific post irradiation, only in spermatogonia and Leydig cells. Interestingly, p53 immunostaining in Sertoli cells increased after irradiation (Figures 1a and b). Nevertheless, mutp53 was still undetectable in spematocytes, spermatids and myoid cells. p53 staining was negative in *p53*^*−/−*^ controls (Figure 1b). This shows that DNA damage-driven p53 accumulation is a consistent mechanism in both p53 wild-type and mutant testicular spermatogonia. The response of various spermatogonia to irradiation is different, with the order of increase in mutp53 being Asg/A1-4 > Int Spermatogonia > Bsg.

There was a pronounced difference in the staining intensities between different types and even among the same type of spermatogonia (Figure 1a, cyan arrows marked low and red arrows marked relatively high mutp53 expression). This may be due to the differential accumulation of mutp53 in distinct cell cycle phases. Co-localization of p53/Aurora B was observed in spermatogonia of unirradiated *p53*^*R172H/R172H*^, irradiated *p53*^*R172H/R172H*^ and irradiated *p53*^*+/+*^ mice by double-immunofluorescent staining (Figure 1d). Unlike IHC, only relatively highly expressed p53 proteins could be detected by this technique. Aurora B is a mitotic checkpoint kinase localizing in chromosomes in G2/M transition.^16^ Co-localization of mutp53/Aurora B in unirradiated *p53*^*R172H/R172H*^ indicates that spermatogonia expressing high levels of mutp53 are mostly in G2/M phase.

### Expression of p53 transcripts in testes of *p53*^*+/+*^ and *p53*^*R172H/R172H*^ mice

**Figure 2:**
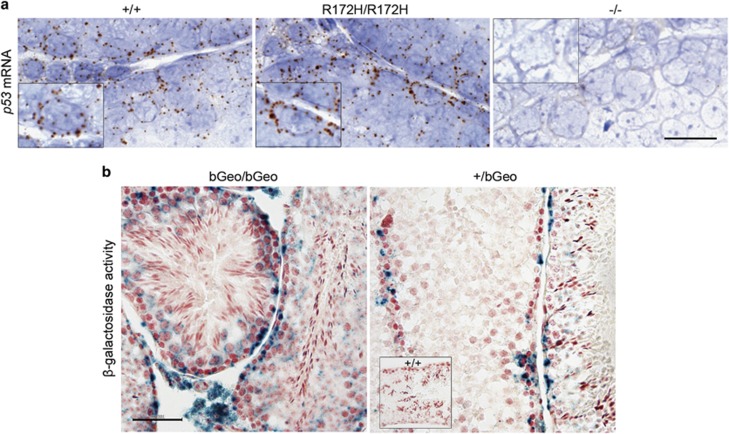
p53 transcripts in *p53*^*+/+*^ and *p53*^*R172H/R172H*^ mice testes. (**a**) p53 mRNA *in situ* hybridization by RNAscope technique in *p53*^*+/+*^, *p53*^*R172H/R172H*^ and *p53*^*−/−*^ mice testes. Inset boxes show magnified view of individual spermatogonium expressing p53 mRNA. Scale bar=20 μm. (**b**) β-galactosidase activity in spermatogonia of adult βGeo homozygous and heterozygous p53 promoter report mice. X-Gal staining was used to determine the βGeo expression patterns in mouse testis. Inset box shows *p53*^*+/+*^ negative control. Scale bar=100 μm.

Detectable wtp53 protein in spermatogonia may indicate high levels of the p53 transcript in these cells. This was confirmed by RNA *in situ* hybridization (ISH), high amounts of p53 mRNA in spermatogonia and Leydig cells of both *p53*^*+/+*^ and *p53*^*R172H/R172H*^ mice were detected (Figure 2a). Although spermatocytes and Sertoli cells did not show detectable p53 protein, RNA ISH was able to detect the presence of an extremely low amount of p53 mRNA. The mRNA levels of p53, as well as protein levels of Mdm2 (Supplementary Figure S2), were similar between *p53*^*+/+*^ and p53^*R172H/R172H*^ mice testes. This may indicate that increased p53R172H levels in spermatogonia are mostly due to the stabilization of mutp53 protein, but not due to an increase in transcription or a decrease in Mdm2 protein levels.

To determine if this is a true positive staining, we generated p53 promoter reporter mice using a gene-trapping approach to measure p53 promoter activity in various testicular cells. A cassette encoding a β-galactosidase (LacZ)-neomycin fusion protein (βGeo) was inserted into intron 1 of the p53 gene sequence, and was under the control of the endogenous p53 promoter. β-galactosidase activity indicated p53 gene transcription and could be visualized by X-gal staining. β-galactosidase was detected in spermatogonia and Leydig cells of *p53*^*βGeo/βGeo*^ and spermatocytes only showed background staining (Figure 2b). Homozygous βGeo mouse testis expressed higher β-galactosidase than heterozygous mice. This was in accordance to our IHC staining for wtp53 protein. This confirms the high p53 transcript levels in spermatogonia and Leydig cells of the *p53*^*βGeo/βGeo*^ testes and indicates that the absence of p53 protein in other cells is a result of epigenetic silencing at the transcription level. It also establishes that p53 transcription is not regulated by p53 itself.

### Expression of mutant p53 in embryonic and postnatal testes of *p53*^*R172H/R172H*^ mice

**Figure 3:**
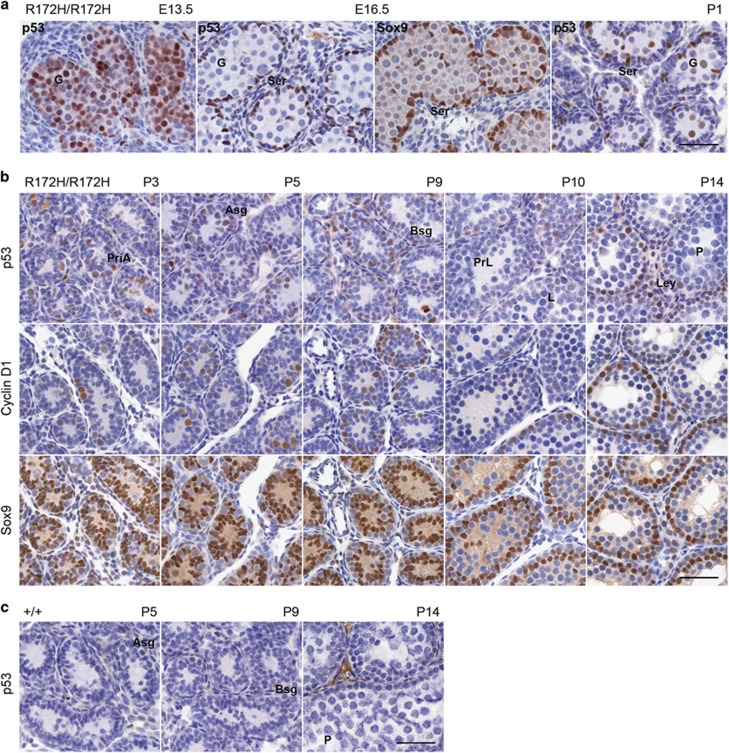
Mutant p53 expression in embryonic and postnatal testes. Time mating was done for analysis of p53 expression in embryonic and postnatal testes to further confirm their expression in different kinds of spermatogonia in *p53*^*R172H/R172H*^ and *p53*^*+/+*^ mice. (**a**) p53 IHC of E13.5, E16.5 embryos and P1 neonate testes from *p53*^*R172H/R172H*^ mice. Sox9 at E16.5 was also stained for identification of pre-Sertoli cells. (**b**) P3, P5, P9, P10, and P14 *p53*^*R172H/R172H*^ pups and (**c**) P5, P9, P14 *p53*^*+/+*^ pups were sacrificed and testes were harvested for p53 IHC analysis. Cyclin D1 and Sox9 were also stained to label spermatogonia and Sertoli cells. PriA: primitive A spermatogonia, Asg: type A_undiff_ spermatogonia, Bsg: type B spermatogonia, PrL: preleptotene, L: leptotene, P: pachytene, Ley: Leydig cell. Scale bars=50 μm.

At a gestational age of 11.5 days (E11.5), primordial germ cells are enclosed by somatic cells (pre-Sertoli cells). Primordial germ cells become gonocytes and proliferate until E15.5 before becoming quiescent. Gonocytes renew proliferation shortly after birth to give rise to Asg.^17, 18^ At E13.5, gonocytes uniformly expressed mutp53. However, at E16.5, gonocytes stopped expressing mutp53 but pre-Sertoli cells marked by Sox9 expressed strong mutp53 (Figure 3a). At postnatal day 1 (P1), mutp53 in gonocytes started to express, whereas its expression in pre-Sertoli cells was diminished (Figure 3a). Strong Ki67 was detected in E13.5 gonocytes and E16.5 pre-Sertoli cells (Supplementary Figure S3a) revealing these mutp53 expressing cells are actively proliferating. In adult mice, Ki67 was not detectable in Sertoli cells but was expressed highly in cycling spermatogonia (Supplementary Figure S3b). This suggests that mutp53 is consistently expressed at a detectable level in proliferating and cycling cells. This is entirely consistent with our earlier studies on mutp53 expression in the intestine.

Given the developmentally timed appearance of the distinct spermatogonial types in seminiferous tubules, the examination of postnatal mouse testis during the first wave of spermatogenesis would enable us to further specify the types of spermatogonia expressing mutp53. Spermatogenesis initiates 2–3 days after birth and at P3, gonocytes migrate to the lamina and become primitive type A spermatogonia.^19^ These cells expressed both mutp53 and cyclin D1 revealing their proliferative properties (Figure 3b, PriA). In contrast, Sertoli cells marked by Sox9 were immunonegative for mutp53 and cyclin D1, which indicates that by P3, Sertoli cells have already become quiescent.

Asg first appeared at P4-5, Bsg and Int spermatogonia could be found in P9 testes. We observed strong mutp53 and cyclin D1 expression in Asg, Int and Bsg. Sox9 positive Sertoli cells were mutp53 and cyclin D1 negative. Pre/leptene (PrL), zygotene (Z), pachytene (P) spermatocytes could be found at P10, P14 but mutp53 was undetectable in these cell types. Mutp53 was also undetectable in secondary spermatocytes at P21 and P28 (data not shown). We also detected the expression of wtp53 in postnatal testes of *p53*^*+/+*^ mice. A weaker expression of wtp53 could be observed in Asg at P5 and Bsg at P9.

The data from developing embryonic/postnatal to adult testes further indicate that mutp53 protein can be detected in proliferating type A, Int and B spermatogonia, a subset of Leydig cells, proliferative pre-Sertoli cells during embryonic stages, proliferating gonocytes and PriA in embryos and neonate.

### Persistent DNA double-strand breaks induced by irradiation in p53R172H mouse testis

As described earlier, mutp53 is not necessary for testicular tumorigenesis.^2^ Therefore, it is important to investigate if the DNA damage response in p53 wild-type testes and mutant testes differs. *p53*^*+/−*^ and *p53*^*R172H/−*^ mice were subjected to 2 Gy irradiation and testes were harvested at different time points including 3 h, 6–8 h, 1 day, 2–3 days and 6 days post irradiation. The induction of p53, p21 and DNA double-strand breaks (DSBs), the states of proliferation and apoptosis were compared in irradiated *p53*^*+/−*^ and *p53*^*R172H/−*^ mice testes by staining of p53, p21, 53BP1, γH2AX, PCNA and cleaved caspase 3.

**Figure 4:**
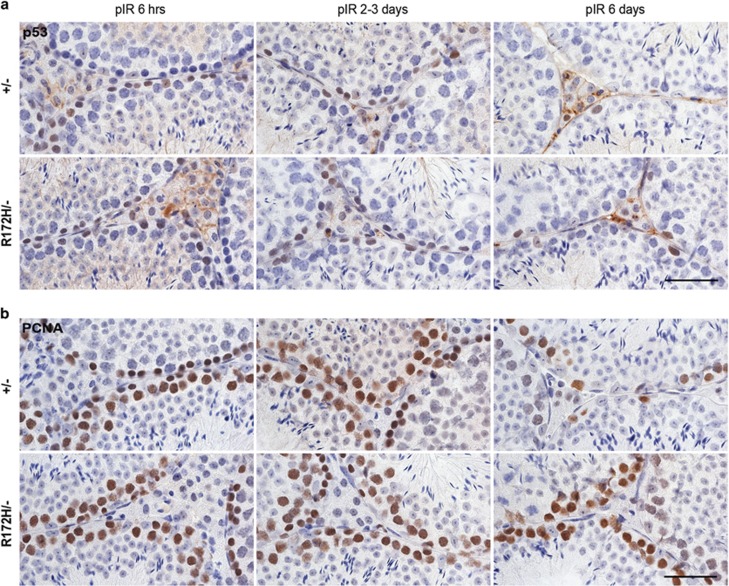
Activation of wild-type and mutant p53 protein by irradiation. *p53*^*+/−*^ and *p53*^*R172H/−*^ mice were irradiated with 2 Gy and sacrificed at indicated time-points. Consecutive testis sections were used for IHC analysis of (**a**) p53 accumulation and (**b**) PCNA immunopositive staining representing proliferative states of various testicular cells at indicated time points. pIR: post irradiation. Scales bars=50 μm.

The basal levels of p53 in *p53*^*+/−*^ and *p53*^*R172H/−*^ mice testes were low (Supplementary Figure S1); however, both wtp53 and mutp53 levels increased as quickly as 3 h and increased stably at 6–8 h and 2–3 days post 2 Gy irradiation (Figure 4a). The number of wtp53 positive cells dropped sharply after 1 day, whereas the number of mutp53-positive cells slightly declined 2–3 days post irradiation. At 6 days post irradiation, the number of spermatogonia was markedly reduced in both *p53*^*+/−*^ and *p53*^*R172H/−*^ mice testes. Proliferation marker PCNA was sharply reduced in testes of *p53*^*+/−*^ mice but kept relatively high in *p53*^*R172H/−*^ mice 6 days post irradiation (Figure 4b). p21 was not induced in spermatogonia of *p53*^*+/−*^ and *p53*^*R172H/−*^ mice testes at any time point post irradiation. However, strong p21 could be observed in adjacent epididymal epithelia in *p53*^*+/−*^ mice and this indicated a successful p21 IHC staining (data not shown).

**Figure 5:**
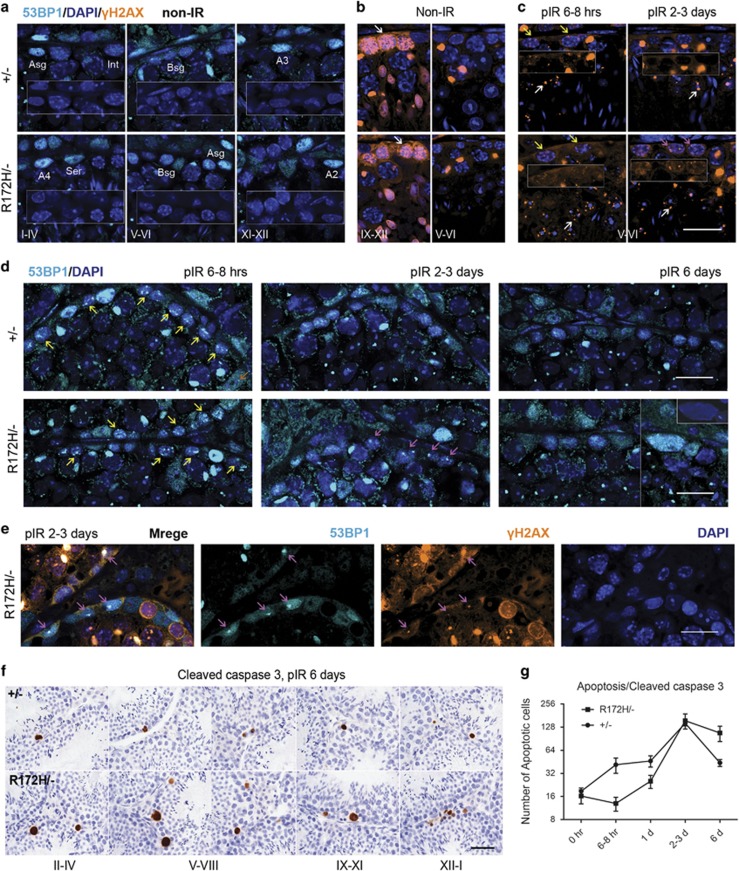
Persistent DNA double-strand breaks and apoptosis induced by irradiation. *p53*^*+/−*^ and *p53*^*R172H/−*^ mice were irradiated with 2 Gy and sacrificed at indicated time points. The testes of non-irradiated control and irradiated mice were harvested for analysis. (**a** and **b**) 53BP1 and γH2AX immunostaining of non-irradiated mice testes. 53BP1 pan-nuclear staining was observed in Asg, A1-A4 spermatogonia. White arrows: γH2AX condensed staining in leptotene and zygotene spermatocytes. Sex-bodies and spermatids expressed high γH2AX. (**c** and **d**) Time course of DSB induction indicated by 53BP1 and γH2AX immnunopositive foci signals. pIR: post irradiation. Yellow arrows: DSBs foci 6–8 h post irradiation. Purple arrows: DSBs foci 2–3 days post irradiation. White arrows: γH2AX foci staining in spermatids. (**e**) 53BP1/γH2AX co-labelling of testes from *p53*^*R172H/−*^ mice 2–3 days post 2 Gy irradiation. Purple arrows: co-expressing of 53BP1/γH2AX in DSBs foci. (**a**–**e**): Scale bars=20 μm. (**f**) Apoptosis induction indicated by cleaved caspase 3 IHC staining. Scale bar=50 μm. (**g**) Quantification of the number of apoptotic cells at various time points of *p53*^*+/−*^ and *p53*^*R172H/−*^ mice by counting cleaved caspase 3 positive cells. Slides were scanned by VSViewer system and 20 views of × 10 images per slide were captured for the counting. Data points represent the mean values for each group.

To understand DSBs induction in testes, we performed immunostaining for phospho-ATM at Ser 1981 (pATM), 53BP1 and γH2AX in testes from *p53*^*+/−*^ and *p53*^*R172H/−*^ mice with and without irradiation. In non-irradiated control mice testes, we observed distinct levels of pan-nuclear distribution of 53BP1 in a subset of spermatogonia (mostly A spermatogonia) representing a pre-existing pool of 53BP1 (Figure 5a). Condensed γH2AX and prominent pATM staining patterns were observed in leptotene, zygotene spermatocytes in both non-irradiated (Figure 5b, white arrows, Supplementary Figure S4a) and irradiated mice testes (data not shown). The increased expression of pATM in these spermatocytes and p53 in spermatogonia may indicate their critical roles in spermatogenesis (Supplementary Figure S4b).

DSBs, indicated by 53BP1 and γH2AX foci staining, were similarly induced in spermatogonia of *p53*^*+/−*^ and *p53*^*R172H/−*^ mice testes at 3 and 6–8 h time points (Figures 5c and d, yellow arrows). DSBs were induced in A1-4, Int, B spermatogonia, preleptotene, Sertoli cells and other cell types, and were more prominent in B spermatogonia. This is consistent with a previous report.^20^ Apoptosis is an important response in testes of both *p53*^*+/−*^ and *p53*^*R172H/−*^ mice to clear cells with DSBs out of the system. In *p53*^*+/−*^ mice, DSBs were resolved 2–3 days post irradiation with a high apoptosis rate during 6–24 h post irradiation. *p53*^*R172H/−*^ mice showed a lower apoptosis efficacy at this time point but presented persistent DSBs expressing both 53BP1 and γH2AX 2–3 days post irradiation (Figures 5e–g). The DSBs in p53R172H mice were also cleared 6 days post 2 Gy irradiation via a late apoptosis event. *p53*^*+/−*^ mice showed two apoptosis peaks at 6–24 h and 2–3 days post irradiation. *p53*^*R172H/−*^ mice showed an increase in the number of apoptotic cells at 2–3 days post irradiation, with the number of these cells remaining higher than *p53*^*+/−*^ cells after 6 day post irradiation (Figure 5g).

### Long-term effects of DSBs induced by irradiation: formation of GSG in *p53*^*R172H/R172H*^ and *p53*^*−/−*^ mice

To further determine the long-term effects of DSBs induced by irradiation, we subjected *p53*^*+/+*^, *p53*^*R172H/R172H*^ and *p53*^*−/−*^ mice to 2 Gy irradiation and harvested the testes 3 h, 14 and 21 days post irradiation. Since the adult seminiferous epithelium repeats a cycle every 8.6 days,^21^ the interval between irradiation and necropsy permits for 2–3 seminiferous epithelium cycles to take place allowing us to observe if (and how) cell cycle progression is impaired differently in mice of distinct genotypes. In addition, this enables us to verify any loss-of-function and/or gain-of-function of p53R172H mutant.

**Figure 6:**
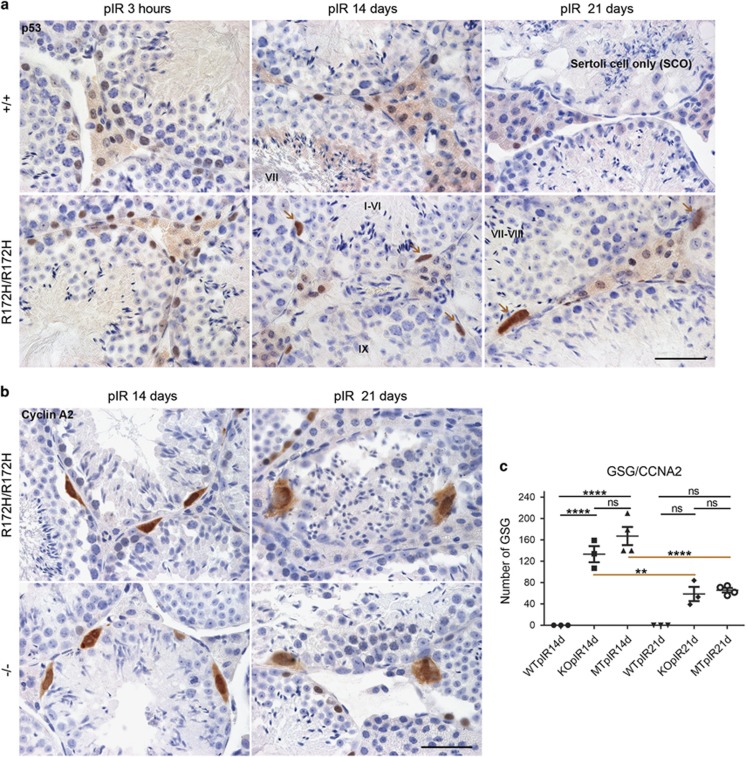
The formation of giant spermatogonia triggered by DSBs in p53R172H and p53-null mice. (**a**) p53 IHC of testes from *p53*^*+/+*^ and *p53*^*R172H/R172H*^ mice irradiated with 2 Gy and sacrificed at 3 h, 14 days and 21 days post irradiation (pIR). Brown arrows: giant spermatogonia (GSG). Scale bar=50 μm. (**b**) Cyclin A2 IHC of testes from *p53*^*R172H/R172H*^ and *p53*^*−/−*^ mice irradiated with 2 Gy and sacrificed at 14 days and 21 days pIR. Scale bar=50 μm. (**c**) Quantification of the number of GSG: GSG were counted as number of cyclin A2 positive cells. Slides were scanned by VSViewer system and 20 views of × 10 images per slide were captured for the counting. The data were analysed by PRISM software and *P*-values were calculated by 1-way ANOVA. The horizontal bars represent ±s.d., **P⩽0.01, ****P⩽0.0001, ns, not significant.

At 14–21 days post irradiation, *p53*^*R172H/R172H*^ mice showed loss of spermatogonia. However, there was an interesting appearance of some dysplastic cells, named ‘Giant spermatogonia' (GSG) lying on the basal compartment of the different stages of seminiferous tubules (Figure 6a). The morphology of GSG in testes of *p53*^*R172H/R172H*^ mice was dysplastic: with irregularly shaped nuclei at least twice the size of typical spermatogonial nuclei. The GSG consistently expressed high levels of p53R172H protein and their size was generally larger at 21 days than at 14 days post irradiation. Such GSG were not found in *p53*^*+/+*^ mice testes 14/21 days post irradiation. However, from 14–21 days post irradiation, *p53*^*+/+*^ testes showed a marked reduction in the number of cells and a wider interstitial gap between seminiferous tubules than that in *p53*^*R172H/R172H*^ mice. In severe cases of *p53*^*+/+*^ testes 21 days post irradiation, seminiferous tubules exhibited histology similar to that seen in Sertoli cell only syndrome, where seminiferous tubules contained only Sertoli cells and lacked spermatogonia.

**Figure 7:**
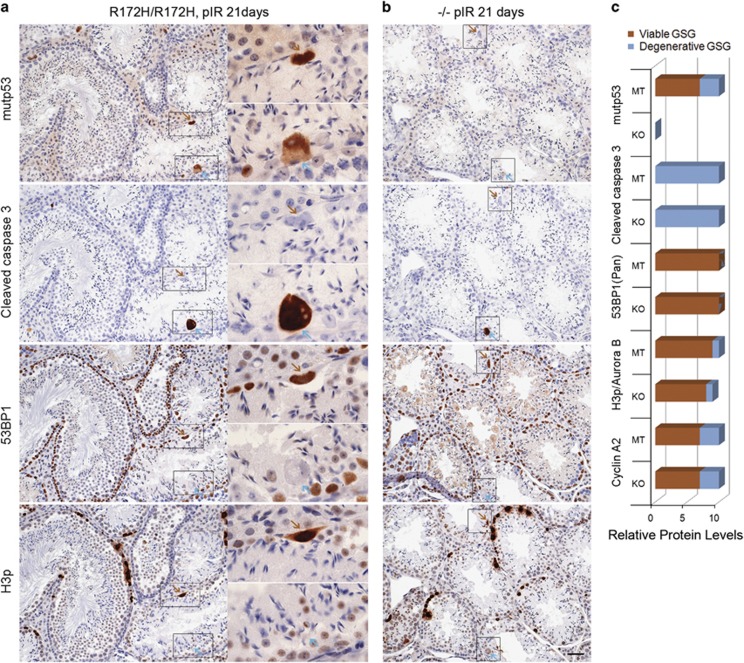
Elimination of Degenerative giant spermatogonia via programmed cell death. (**a**) *p53*^*R172H/R172H*^ and (**b**) *p53*^*−/−*^ mice were irradiated with 2 Gy and sacrificed at 21 days. The testes were harvested for IHC analysis in consecutive sections for p53, cleaved caspase 3, 53BP1 and H3p. Brown arrows: Viable giant spermatogonium from one cell in four sections; Cyan arrows: Degenerative giant spermatogonium from another cell in four sections. Scale bar=50 μm. (**c**) Relative protein levels in two kinds of GSG were summarized. Brown columns: Viable GSG; cyan columns: Degenerative GSG. Relative protein levels ranged from 1 to 10 according to the observation of individual protein expression level in testes.

Similar GSG could be found in irradiated *p53*^*−/−*^ mice (Figures 6b and 7b). To better visualize the GSG in *p53*^*−/−*^ and *p53*^*R172H/R172H*^ mice, cyclin D1 was stained as we did in developing testes. However, cyclin D1 was immnunonegative in these GSG (data not shown) indicating they were no longer actively proliferating.^22^ We then stained for cyclin A2 and Aurora B, which were immunopositive in mutp53 highly expressed G2/M phase spermatogonia (Figure 1d). Strikingly, both cyclin A2 and Aurora B expressed in almost all GSG with heterogeneous staining intensities (Figure 6b, Supplementary Figure S5a) indicating these GSG were blocked in G2/M phase asynchronously. The consistent expression of cyclin A2 in GSG of both *p53*^*−/−*^ and *p53*^*R172H/R172H*^ mice testes enables the precise quantification of these GSG. At 14 and 21 days post irradiation, *p53*^*R172H/R172H*^ and *p53*^*−/−*^ mice showed greater frequencies of GSG (*P*<0.05) compared to *p53*^*+/+*^ mice. Although we could not find significant differences in the number of GSG between *p53*^*R172H/R172H*^ and *p53*^*−/−*^ mice 14/21 days post irradiation, the sharply reduced number of GSG at 21 days compared to that at 14 days post irradiation indicates those GSG might have been eliminated from system by programmed cell death (Figure 6c).

### Subsets of GSG were eliminated via programmed cell death

To determine the consequences of the GSG formed in *p53*^*R172H/R172H*^ and *p53*^*−/−*^ mice post irradiation, we detected cleaved caspase 3 with Aurora B (Supplementary Figure S5a) and cleaved caspase 3 with p53, 53BP1, phospho-Histone H3 (H3p) in consecutive testis sections from mice 21 days post irradiation (Figure 7). The huge size of GSG made it easier to observe the co-expression of these proteins in the same giant spermatogonium. Two distinct GSG were observed in *p53*^*R172H/R172H*^ (as well as in *p53*^*−/−*^ mice that were identical, except for GSG in *p53*^*−/−*^ mice that did not express mutp53): (1) Viable GSG expressed high level of mutp53, cyclin A2, pan-nuclear 53BP1 distribution and condensed H3p/Aurora B. Furthermore, viable GSG did not co-express cleaved caspase 3 and thus were not apoptotic (Figure 7, Supplementary Figure S5a, brown arrows). (2) Degenerative GSG showed weaker mutp53, cyclin A2 and H3p/Aurora B staining, but co-expressed a high level of cleaved caspase 3 and were negative for pan-nuclear 53BP1 indicating their degenerative nature and propensity to die by programmed cell death (Figure 7, Supplementary Figure S5a, cyan arrows).

At 14 days post irradiation, *p53*^*R172H/R172H*^ showed a higher number of apoptotic cells than *p53*^*+/+*^. However, other comparisons between *p53*^*+/+*^ and *p53*^*−/−*^, *p53*^*R172H/R172H*^ and *p53*^*−/−*^ mice at 14/21 days post irradiation failed to show significant apoptosis differences (Supplementary Figure S5b). However, there was a general trend towards increased apoptotic level in *p53*^*R172H/R172H*^ >*p53*^*−/−*^ >*p53*^*+/+*^. Alternatively, it is possible that apoptotic waves might have occurred before 14 days post irradiation (Figure 5g).

### p53R172H promotes spontaneous formation of GSG in p53R172H mice

**Figure 8:**
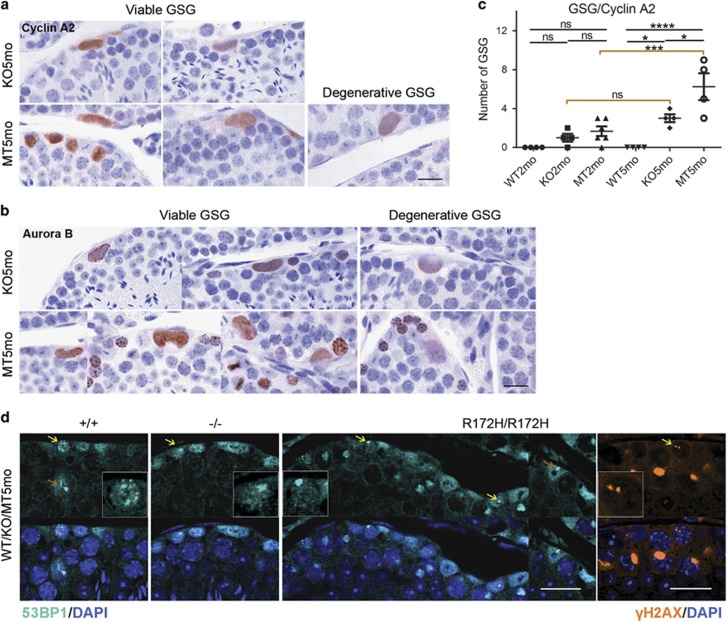
Spontaneous formation of giant spermatogonia in *p53*^*R172H/R172H*^ ageing mice. (**a** and **b**) IHC localization of cyclin A2 and Aurora B in spontaneously formed GSG in ageing *p53*^*R172H/R172H*^ and *p53*^*−/−*^ mice. Viable and Degenerative GSG are indicated. Heterogeneous expression of two proteins in GSG represents gradually degenerating status of the GSG. Scale bars=20 μm. (**c**) Quantification of the number of GSG: GSG were counted as number of cyclin A2 positive cells in testes of 2-mo and 5-mo of *p53*^*+/+*^, *p53*^*R172H/R172H*^ and *p53*^*−/−*^ mice. Slides were scanned by VSViewer system and 20 views of × 10 images per slide were captured for the counting. The data were analysed by PRISM software and *P*-values were calculated by 1-way ANOVA. The horizontal bars represent ±s.d., *P⩽0.05, ***P⩽0.001, ****P⩽0.0001, ns, not significant. (**d**) Spontaneous DSBs increased in ageing mice. 53BP1 positive foci representing DSBs were detected in 5-mo testes of *p53*^*+/+*^, *p53*^*R172H/R172H*^ and *p53*^*−/−*^ mice. γH2AX was also detected in 5-mo testes of *p53*^*R172H/R172H*^ mice. Large DSBs foci were consistently found in *p53*^*R172H/R172H*^ mice testes. Scale bars=20 μm.

The formation of GSG was not limited to irradiated *p53*^*R172H/R172H*^ and *p53*^*−/−*^ mice. Similar GSG were present in unirradiated 2 and 5 months old (-mo) *p53*^*R172H/R172H*^ and *p53*^*−/−*^ mice but absent in *p53*^*+/+*^ mice indicated by the immunostaining of cyclin A2, Aurora B and mutp53 (Figures 8a and b; Supplementary Figure S6a). Since >90% of p53-null or p53-mutant mice die by 6-mo as a result of lymphomas,^23, 24^ 5-mo mice were considered ageing mice in this study. Different stages of Viable and Degenerative GSG were also observed and the frequency of GSG in both *p53*^*R172H/R172H*^ and *p53*^*−/−*^ mice was counted according to cyclin A2 positive cells. The number of GSG in *p53*^*R172H/R172H*^ mice increased as mice age from 2 to 5-mo (*P*<0.05) (Figure 8c). At 5-mo, the number of GSG in *p53*^*R172H/R172H*^ mice was significantly higher when compared with that in *p53*^*−/−*^ mice (*P*<0.05), indicating that p53R172H could possibly promote the formation of GSG.

The gross increase in the frequency of GSG in irradiated mice suggests a possible role of DSBs in their formation. Therefore, we evaluated spontaneous DSBs in *p53*^*+/+*^, *p53*^*R172H/R172H*^ and *p53*^*−/−*^ mice testes by 53BP1 and γH2AX immunostaining. Although DSBs were rare in younger 2-mo mouse testis, there was a general increase in spermatogonia and Sertoli cells of 5-mo *p53*^*+/+*^, *p53*^*R172H/R172H*^ and *p53*^*−/−*^ mice (Figure 8d). The number of DSB foci was difficult to quantify due to special characteristic of testes. However, the size of DSB foci indicated in *p53*^*R172H/R172H*^ spermatogonia was relatively larger and more consistently found and these foci were not clustered in nucleolar regions (Supplementary Figure S6b), suggesting a failure of DSB repair in *p53*^*R172H/R172H*^ mouse spermatogonia. If this is the case, p53R172H promotes the formation of GSG via induction, exacerbation or maintenance of DSBs.

## Discussion

Previous studies on the expression of p53 protein in mouse testis have shown mixed results. Almon *et al* showed p53 mRNA and protein expression only in primary spermatocytes.^25^ Beumer *et al* found that in non-stressed mouse testis, pachytene spermatocytes in stages XI–VIII were lightly stained for p53 by IHC, and after irradiation, all spermatogonia and preleptotene spermatocytes in stage VII stained heavily for p53.^26^ Our current findings show mutp53 expression in A_undiff_, A_diff_, Int and B spermatogonia and a subset of a Leydig cells but absent in any type of spermatocytes. wtp53 was also observed in spermatogonia and the intensity of wtp53 staining is generally weaker than that of p53R172H. However, their similar staining patterns indicate that the expression of p53R172H can be a surrogate marker of p53 transcription. Both wtp53 and mutp53 levels increased greatly after irradiation but consistently confined to spermatogonia and Leydig cells. Our results from RNA ISH and p53 promoter reporter mice further confirmed high levels of p53 transcript in spermatogonia. This suggests that genetic silencing at the level of transcription may be responsible for the lack of p53 expression in other cell types, for example spermatocytes. Efficient transcription capacity and high p53 protein levels in spermatogonia provide new evidence for understanding normal spermatogenesis and the development of testicular cancers.

Sertoli cells are supportive cells for nurturing male germ cells and forming a blood-testis barrier to protect the germ cells from invasion.^27^ Our data showed weak/negligible mutp53 but higher Mdm2 in unirradiated Sertoli cells. However, p53 accumulation was observed in Sertoli cells post irradiation. Ahmed *et al* found that Sertoli cells can renew proliferation when isolated from the adult mouse/human testes in culture and they suggested Sertoli cells more resemble arrested proliferating cells.^28^ Our observation of positive p53 staining in adult Sertoli cells post irradiation differs from that seen in typical quiescent somatic cells, such as Paneth cells in the small intestine in which mutp53 expression remains absent even post irradiation.^6^ Absence of detectable p53 protein in Sertoli cells under unirradiated conditions may due to high levels of Mdm2 (Supplementary Figure S2).

Our time-course of irradiation shows that both wtp53 and mutp53 accumulated quickly post irradiation; however, the high levels of p53 in both *p53*^*+/−*^ and *p53*^*R172H/−*^ testicular cells after irradiation did not induce p21 expression. Apoptosis was massively induced and seems to be the main DNA damage response mechanism to clear DSB-ridden cells from the system. In *p53*^*+/−*^ mouse testis, marked apoptosis occurred early at 6–24 h post irradiation; this was not seen in *p53*^*R172H/−*^ mice. Therefore, wtp53 confers mice with an enhanced ability to respond to DNA damage via p53-dependent apoptosis more quickly. This accounts for the greater radiosensitivity of spermatogonia in p53 wild-type mice. Late apoptosis events were present in both *p53*^*+/−*^ and *p53*^*R172H/−*^ mice testes at/after 2–3 days post irradiation, suggesting the significance of p53 independent apoptosis in preventing the persistence of damaged cells in both *p53*^*+/−*^ and *p53*^*R172H/−*^ mice testes. This could explain the failure of mutp53 to induce tumorigenesis in testes with a greater efficacy.

The induction of GSG by irradiation in *p53*^*R172H/R172H*^ and *p53*^*−/−*^ mice but not in *p53*^*+/+*^ mice suggests that the loss of p53 function seems to be a requirement for the formation of GSG. Furthermore, as the number of GSG hugely increased in irradiated *p53*^*R172H/R172H*^ and *p53*^*−/−*^ mice testes, DNA damage seems to play a role as a promoting trigger. It remains unclear how exactly DNA damage promotes the formation of GSG. Given that a proportion of GSG appears bi-nuclear or in pairs, we suspect that the loss of wtp53 function allows spermatogonia to be released from the G2/M checkpoint prematurely in the presence of DNA damage, which may lead to abortive cell division or cytokinesis and further induce GSG. Furthermore, unlike other epithelia, spermatogonial cells are unable to put senescence into effect under circumstances of DNA damage, possibly due to lack of p21. Under the principle of ‘divide or apoptosis', DSBs can accumulate in spermatogonia until apoptosis occurs in absence of wtp53. Meanwhile, continued DNA replication or impaired spermatogonial division leads to GSG.

The higher frequency of spontaneous/unprovoked formation of GSG in unirradiated *p53*^*R172H/R172H*^, relative to *p53*^*−/−*^ mice testes, and the evidence of more pronounced and severe DSBs in unirradiated p53^*R172H/R172H*^ suggests a gain-of-function effect of p53R172H mutant. p53R172H may induce spontaneous DSBs or exacerbate endogenous DSBs, which seems to be a direct reason for the formation of GSG. The enhanced ability of p53R172H to promote the formation of GSG could also be mediated by Aurora B Kinase, which was overexpressed in mutp53 positive spermatogonia and GSG (Figure 8b). Dysregulation of Aurora B kinase is a possible reason for impaired cell division and the induction of DSBs; however, further studies would be required to confirm this.

Giant cells are frequently detected in testicular germ cell tumours^13, 29^ and other tumour^30^ and cancer types.^31^ Beumer *et al* have reported the presence of giant cells in p53-null testes, suggesting that giant cells eventually degenerate via apoptosis over a long time period.^26^ Rotter *et al* suggested that the lack of DNA repair in p53-null mice causes incomplete cytokinesis of tetraploid primary spermatocytes leading to degenerative giant cell formation.^32^ Unlike other reports that the giant cells are mostly derived from spermatocytes in p53-null mice, the GSG we identified originated from spermatogonia, which further elucidates the important role of p53 in spermatogonia. Furthermore, our study is the first to distinguish and characterize Viable and Degenerative GSG seen in p53^*R172H/R172H*^ and *p53*^*−/−*^ mice. We have also shown that the process of GSG degeneration is accompanied by the presence of a delayed onset of p53-independent apoptosis. Our data may suggest a possible reason why testicular cancers do not harbour mutated p53. Further studies would be necessary to determine if all Viable GSG eventually degenerate or become precancerous. Characterizing GSG is important in understanding their incidental findings in testicular biopsies. Exploring mechanisms of late-onset p53-independent apoptosis would be important for discovery of new therapeutic targets to clear dysplastic cells out of the system.

## Materials and methods

### Mice

All mouse experiments were approved by A*STAR Institutional Animal Care and Use Committee (IACUC) and performed in compliance with IACUC regulations. The murine Trp53 knockout and p53R172H mice and the irradiation methods to those mice were previously described.^6^ p53 promoter reporter mice was generated using gene-trapping approach (see Supplementary Information). Briefly, β-galactosidase (LacZ) and neomycin (neo) resistance gene cassette was inserted into intron 1 of *p53* and allows the expression of β-galactosidase controlled by the endogenous p53 promoter.

### Histology, immunostaining and microscopy imaging

Mice testes for IHC and immunofluorescence staining were proceeded as described previously.^33, 34^ The experiments were repeated three times. The antibodies used for IHC/immunofluorescence staining are listed in Supplementary Information. Specifically, antigen retrieval was carried out by heating slides in a pressure cooker (121 °C) for 10 min in a citrate buffer pH9.0 (S2367, DAKO, Glostrup, Denmark) for p53 staining and pH6.0 (S2369, DAKO) for other antibody staining. The peroxidase-conjugated secondary antibodies used were mouse/rabbit EnVisionC (DAKO) for HRP-immunostaining or anti-rabbit/mouse Alexa 488/568 IgG (Invitrogen, Carlsbad, CA, USA) for immunofluorescence staining. Images were captured with a Zeiss AxioImager upright microscope and Olympus FV1000 upright confocal microscope.^34^

### RNA ISH

Tissue sections were processed for RNA *in situ* detection using the RNAscope Detection Kit and Trp53 (NM_010195) probe according to the manufacturer's instructions (Advanced Cell Diagnostics, Hayward, CA, USA).

### X-Gal staining

Mice testes for X-Gal staining were fixed and stained for X-Gal (5-bromo-4-chloro-3-indolyl-β-D-galactopyranoside) (Invitrogen) as described previously^35^ and see Supplementary Information.

### Protein extraction and western blot

See Supplementary Information.

### Statistical analyses

Four to six mice per group were used for histological analysis. Cyclin A2 and cleaved caspase 3 stained testis slides were scanned by VSViewer system and 20 views of × 10 images were captured for the counting of GSG and apoptosis cells. The *P*-values were analysed using Graphpad software (Prism) and calculated by 1-way ANOVA.
