# Supplementary figures and images for: The hot-spot p53R172H mutant promotes formation of giant spermatogonia triggered by DNA damage
Source: Oncogene. 2016 Nov 21;36(14):2002–13. doi: 10.1038/onc.2016.374 (PMC5390101; doi:10.1038/onc.2016.374)

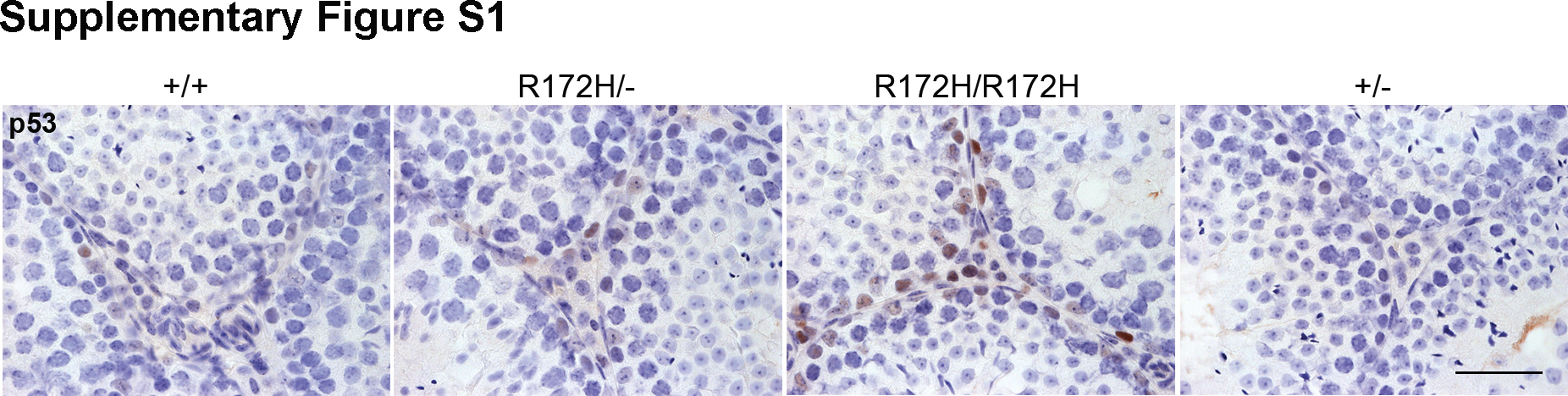

Supplement: Supplementary Figure S1 [file onc2016374x1.tif]

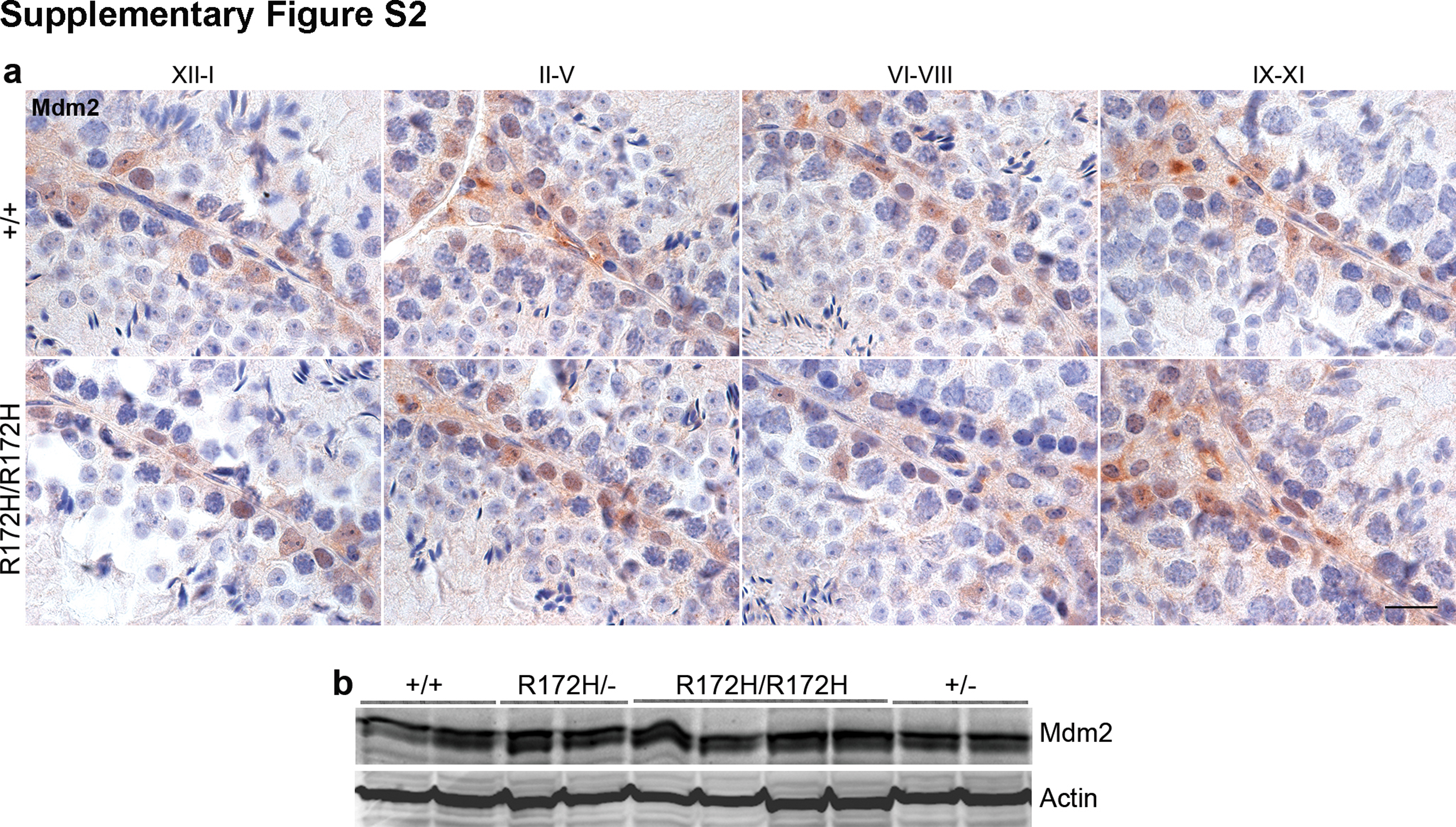

Supplement: Supplementary Figure S2 [file onc2016374x2.tif]

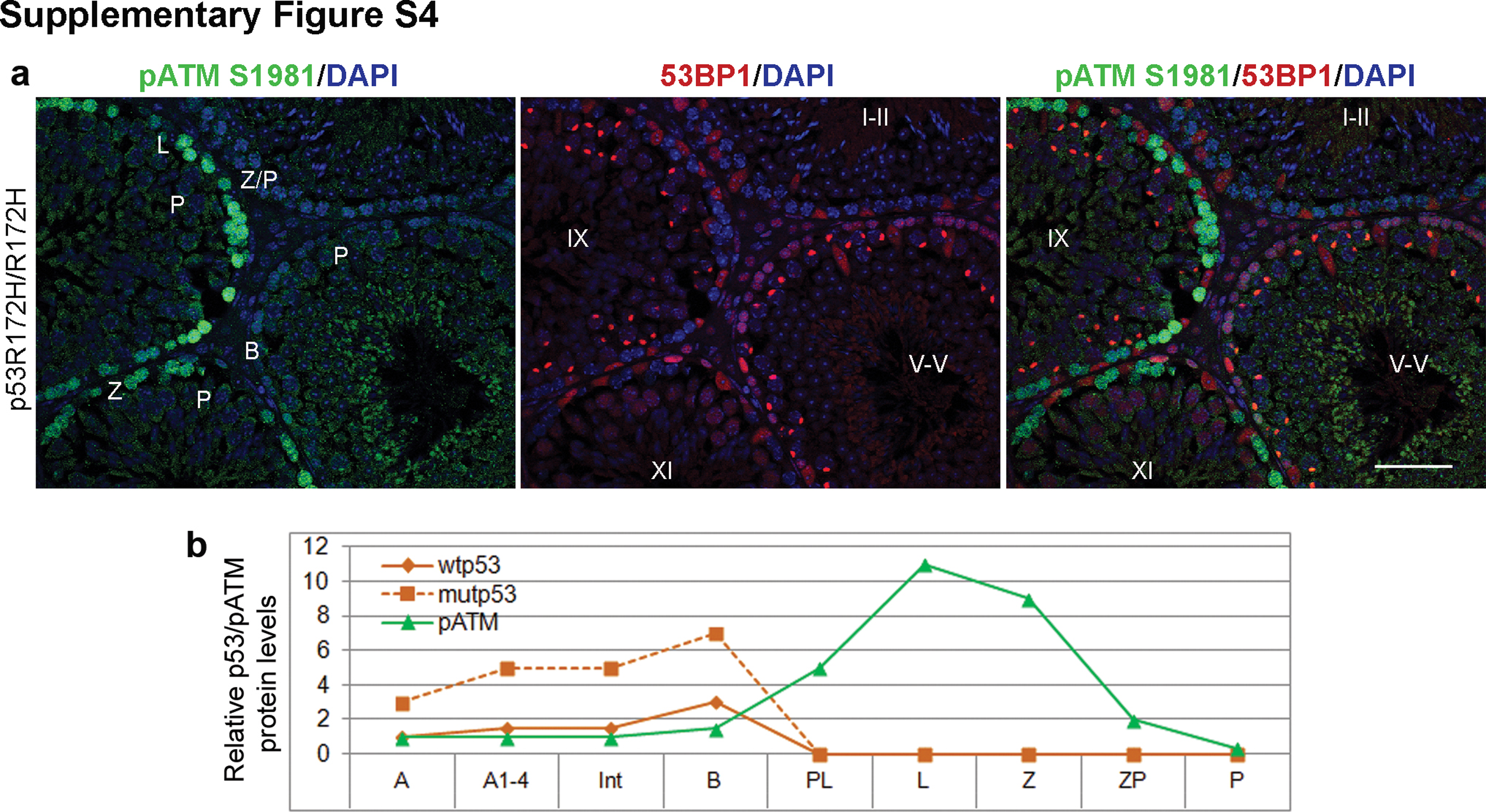

Supplement: Supplementary Figure S4 [file onc2016374x4.tif]

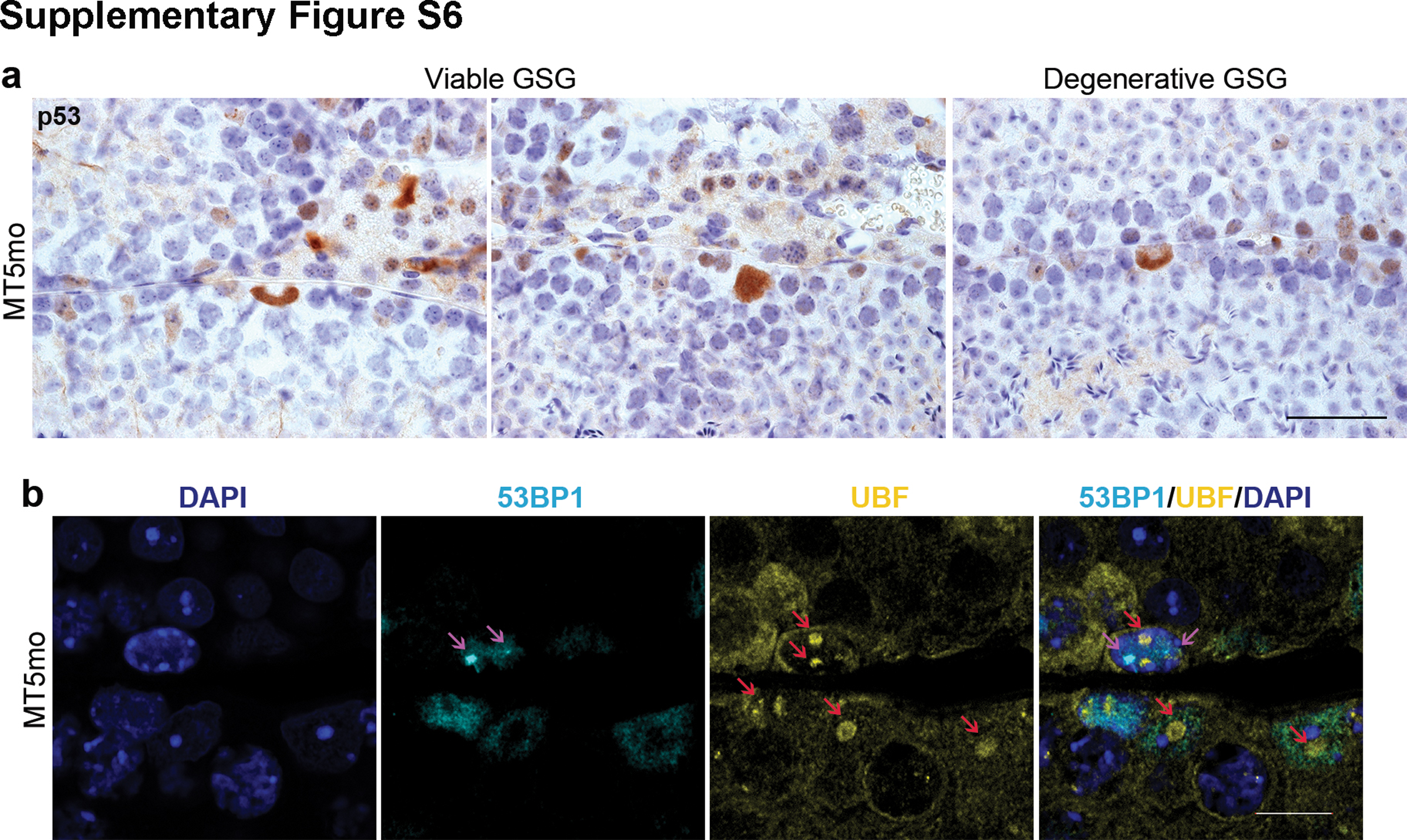

Supplement: Supplementary Figure S6 [file onc2016374x6.tif]
