# Supplementary material for: The hot-spot p53R172H mutant promotes formation of giant spermatogonia triggered by DNA damage
Source: Oncogene. 2016 Nov 21;36(14):2002–13. doi: 10.1038/onc.2016.374 (PMC5390101; doi:10.1038/onc.2016.374)
Supplement: Supplementary Information [file onc2016374x7.docx]

**SUPPLEMENTARY INFORMATION**

### Mice

p53 promoter reporter mouse model was generated using gene-trapping approach. β-galactosidase (LacZ) and neomycin (neo) resistance gene cassette was inserted into intron 1 of *trp53* and the insertion deletes majority of *trp53* gene from intron 1 which creates a trp53-null mutation and allows the expression of β-galactosidase controlled by the endogenous p53 promoter. Mutated mouse embryonic stem cells were microinjected into blastocysts to generate chimeras, from which germline transmission of the null allele of the trp53 gene was accomplished. The target event was confirmed by sequencing and RT-PCR both in ES cell and in chimeras mice. PCR assay was designed to genotype the mice with below primers:

Forward 5’-CACAGTGTCCAGACCATACATAG-3’

Reverse 5’-GCTAAGTAGCCCAAGTTACCTC-3’ (trp53 wild-type allele, 377 bp)

Reverse 5’-AGTGAGGCTTCTAGGACAAGAG-3’ (LacZ-Neo allele, 575 bp)

**X-Gal staining**

Mouse testes for X-Gal staining were fixed in cold 0.2% glutaraldehyde, 2% formaldehyde, 5 mM EGTA (ethylene glycol tetraacetic acid) and 2 mM MgCl2 in 1× phosphate-buffered saline, cold 4% paraformaldehyde in phosphate buffered saline (PBS) for 15 min and stained for X-Gal (5-bromo-4-chloro-3-indolyl-β-D-galactopyranoside) at room temperature overnight. Stained testes were dehydrated and embedded in paraffin for sectioning and counterstained with neutral red (Sigma).

### Protein extraction and western blot

Mouse testes were homogenized and lysed using RIPA buffer (25mM Tris•HCl pH 7.6, 150mM NaCl, 1% NP-40, 1% sodium deoxycholate, 0.1% SDS) supplemented with protease inhibitor cocktail (Roche). Supernatants were collected after centrifugation at 14,000 r.p.m for 1 hour. Protein concentration was determined by the BCA method (Pierce, Thermo Scientific). Proteins were subjected to SDS−PAGE and immunoblot analysis. Blots were probed sequentially with primary and secondary antibodies at the following dilutions: anti-p53 at 1:1000 (1C12, Cell Signaling), anti-Mdm2 at 1:1000, anti-Actin at 1:5000 (Sigma). Secondary HRP-conjugated anti-mouse and anti-rabbit were used at 1:10 000 (GE Healthcare, Chalfont St Giles, UK). Proteins were detected by ECL substrate (Amersham Bioscience) and chemiluminescence was visualized by STORM imaging system (Amersham).

**Antibody list for IHC and WB**

| **Protein** | **Resource** | **Clone** | **Cat No.** | **Brand** | **IHC/IF** | **WB** |
| --- | --- | --- | --- | --- | --- | --- |
| **p53** | Ms | 1C12 | #2425S | Cell Signaling | 1:200 | 1:1000 |
| **Aurora B** | Ms |  | 611082 | BD Transduction | 1:100 |  |
| **Mdm2** | Ms | 2A10 | ab16895 | Abcam | 1:100 | 1:1000 |
| **Cyclin D1** | Ra |  | #2978 | Cell Signaling | 1:100 |  |
| **Sox9** | Ra |  | ab5535 | Abcam | 1:500 |  |
| **Ki67** | Ms | MM1 | NCL | Leica | 1:300 |  |
| **PCNA** | Ra |  | Sc7907 | Santa Cruz | 1:300 |  |
| **53BP1** | Ra |  | NB100-304 | Novus | 1:1000 |  |
| **γH2AX S139** | Ra |  | NB100-2280 | Novus | 1:500 |  |
| **γH2AX S139** | Ms | 2F3 | NB100-78356 | Novus | 1:200 |  |
| **pATM S1981** | Ms |  | #4526 | Cell Signaling | 1:100 |  |
| **UBF** | Ms | F9 | Sc-13125 | Santa Cruz | 1:100 |  |
| **Cleaved caspase 3** | Ra |  | #9661 | Cell Signaling | 1:100 |  |
| **Cyclin A2** | Ra | Y193 | NB110-56899 | Novus | 1:100 |  |
| **H3p (S10)** | Ra |  | ab7031 | Abcam | 1:500 |  |
| **Actin** | Ra |  | A2066 | Sigma |  | 1:5000 |

**SUPPLEMENTARY FIGURE LEGENDS**

**Supplementary Figure S1. Dosage dependent p53 expression in spermatogonia.**

p53 IHC of testes from mice carrying one or two wild-type *p53* or *p53R172H* alleles ( *p53^+/+^*, *p53^R172H/-^*, *p53^R172H/R172H^*, and *p53^+/-^*).

**Supplementary Figure S2. Mdm2 expression in *p53^+/+^* and *p53^R172H/R172H^* mouse testes.**

**a**. Mdm2 IHC of testes from adult *p53^+/+^* and *p53^R172H/R172H^* mice. The stages of the seminiferous epithelium cycles are indicated with Roman numerals. Scale bars = 20 µm. **b**. Western blot analysis of endogenous Mdm2 in testes of various p53 genotypes. Actin was detected as a loading control.

**Supplementary Figure S3**. **Ki67 expression in embryonic and postnatal testes.**

**a**. HE and Ki67 immunostaining of sections of E13.5 and E16 embryos. Centrally located gonocytes surrounded by pre-Sertoli cells are indicated by HE staining. Ki67 was immunostained as a proliferative marker and nuclei were co-stained with DAPI. Scale bars = 50 µm. **b**. Immunostaining of Ki67 in testis sections of adult *p53^R172H/R172H^* mice. Nuclei were co-stained with DAPI. White arrows: Sertoli cells. Red arrow: cycling type A spermatogonia. Yellow arrow: quiescent type A spermatogonia. Ser: Sertoli cell, A_undiff_: A_undiff_ spermatogonia, A_diff_: A_diff_ spermatogonia. Scale bar = 20 µm.

**Supplementary Figure S4. Phospho-ATM expression in testes**

**a**. Co-labeling of pATM and 53BP1 in unirradiated *p53^R172H/R172H^* mouse testis sections. pATM is highly expressed in leptotene and zygotene of prophase I. Scale bar = 50 µm. **b**. Expression patterns of wtp53, mutp53 and pATM proteins in unirradiated mouse testes. Relative protein levels were observed and summarized on a scale of 1-12. There was heterogeneous expression of wtp53 and mutp53 even in the same type of spermatogonia, the graph only indicates relative and average protein levels. wtp53 and mutp53 expression indicated by orange and orange dash respectively, green line represents pATM expression.

**Supplementary Figure S5. Partially colocalization of Aurora B and Cleaved caspase 3 in giant spermatogonia**

**a**. *p53^R172H/R172H^* and *p53^-/-^* mice were irradiated with 2 Gy and sacrificed at 14 and 21 days. The testes were harvested for IHC analysis. Aurora B and cleaved caspase 3 were stained in serial sections. Brown arrows: Viable GSG. Cyan arrows: Degenerative GSG. Scale bar = 50 µm. **b**. Quantification of the number of apoptotic cells by counting the number of cleaved caspase 3 positive cells. Slides were scanned by VSViewer system and 20 views of 10x images per slide were captured for the counting. The data was analysed by PRISM software and *p*-values were calculated by 1-way ANOVA. The horizontal bars represent ± SD, * *P*<0.05, ns, not significant.

**Supplementary Figure S6. Mutant p53 expression in spontaneously formed giant spermatogonia in *p53^R172H/R172H^* ageing mice.**

**a**. IHC localization of mutp53 in testis sections from ageing *p53^R172H/ R172H^* mice (5 months old), different levels of mutp53 expression was observed in Viable and Degenerative GSG. Scale bar = 50 µm. **b.** 53BP1/UBF co-labelling of testes from *p53^R172H/R172H^* mice. Purple arrows: DSBs foci. Red arrows: nucleoli. Scale bar = 10 µm.
